# Screening performance of C-reactive protein for active pulmonary tuberculosis in HIV-positive patients: A systematic review with a meta-analysis

**DOI:** 10.3389/fimmu.2022.891201

**Published:** 2022-08-25

**Authors:** Andreea-Daniela Meca, Adina Turcu-Stiolica, Maria Bogdan, Mihaela-Simona Subtirelu, Relu Cocoș, Bogdan Silviu Ungureanu, Beatrice Mahler, Catalina-Gabriela Pisoschi

**Affiliations:** ^1^ Department of Pharmacology, University of Medicine and Pharmacy of Craiova, Craiova, Romania; ^2^ Department of Pharmacoeconomics, University of Medicine and Pharmacy of Craiova, Craiova, Romania; ^3^ Department of Medical Genetics, University of Medicine and Pharmacy “Carol Davila”, Bucharest, Romania; ^4^ Marius Nasta Institute of Pneumology, Bucharest, Romania; ^5^ Research Center of Gastroenterology and Hepatology, University of Medicine and Pharmacy of Craiova, Craiova, Romania; ^6^ Pneumology Department (II), University of Medicine and Pharmacy “Carol Davila”, Bucharest, Romania; ^7^ Department of Biochemistry, University of Medicine and Pharmacy of Craiova, Craiova, Romania

**Keywords:** tuberculosis, HIV-positive, C-reactive protein, screening, sensitivity, specificity

## Abstract

**Background:**

Tuberculosis (TB) is the leading infectious cause of mortality worldwide. In the last years, resistant strains of the etiological agent, *Mycobacterium tuberculosis*, have emerged, thus demanding more triage tests to identify active pulmonary TB (PTB) patients and to evaluate their disease severity. Therefore, acute-phase reaction serum tests are required for monitoring TB patients, among WHO symptom screening recommendations. C-reactive protein (CRP) is a non-specific inflammatory biomarker that has been recently proposed for TB screening and can be quantitatively analyzed through cost-effective point-of-care assays. A previous meta-analysis found CRP to be highly sensitive and moderately specific for active PTB with confirmed HIV infection.

**Methods:**

We performed a meta-analysis update of diagnostic tests, pooling sensitivities, and specificities in order to assess the accuracy of CRP as a potential test for the screening of HIV-associated PTB in outpatients. We searched MEDLINE, Web of Science, and SCOPUS for eligible articles before 19 October 2021.

**Results:**

We identified 13 eligible studies with HIV-positive patients with PTB. At a CRP threshold of 10 mg/L, CRP pooled sensitivity was 87% (76%–93%) and pooled specificity was 67% (49%–81%), with an area under the curve (AUC) of 0.858. Using a CRP threshold of 8 mg/L, pooled sensitivity was 82% (72%–89%) and pooled specificity was 82% (67%–92%), with an AUC of 0.879. We found that CRP has a high sensitivity in the screening of PTB in HIV-positive outpatients, consistent with findings reported previously.

**Conclusions:**

Regardless of pooled specificity, better results were found using the CRP threshold of 8 mg/L as a test screening of PTB, meeting the need of further approaching specific TB diagnostic methods and reducing resource consumption.

## Introduction

The evaluation of tuberculosis (TB) is necessary in order to achieve the World Health Organization’s (WHO) TB strategy targets. Although WHO expects a reduction in the epidemic TB incidence rate of 90% by 2035 compared with 2015 (which means less than 10 new cases/100,000 individuals), unfortunately, in 2018, the burden of this infectious disease was still high (1, 2). The total global TB incidence in 2018 was 132 new cases/100,000 individuals (7,253,116 new and relapsed TB notified cases), especially in HIV-positive patients (which represented 64% from total number of cases) (1, 2). In 2020, the global TB incidence was 127 new cases/100,000 population (9,870,000 individuals including 157,817 people diagnosed with drug-resistant TB), which represented one infected person every 3 s (1, 2). Even more, an increase in TB mortality rates was noted in 2020 (as 1.3 million deaths among HIV-negative patients and an additional 214,000 among HIV-positive patients were declared in comparison with 1.2 million and 209,000 declared in 2019, respectively) due to the COVID-19 pandemic, which further involved reduced access to hospitals and pharmacotherapy (3). Even though efforts of improving TB care services have been made in the last years, this major infectious disease remains a worldwide threat, difficult to early diagnose and detect, generating high public transmission rates and mortality (4). Since 2014, it has exceeded HIV, becoming one of the top 10 causes of deaths globally (5). The immediate priority claimed by WHO is to restore access to essential services such as anti-TB treatment, preventive measures, drug-susceptibility testing, and rapid case detection through systematic screening (3).

The performance of adequate laboratory methods in the diagnosis of pulmonary tuberculosis (PTB) depends on many factors. First of all, sputum quality and quantity can have an impact on the yield of TB diagnostic results obtained from the microscopic examination of *Mycobacterium tuberculosis* (*M.tb.*) through the Ziehl–Neelsen method and a culture-based technique (6, 7). On the other hand, *M.tb*. requires an elaborated clinical plan in order to be correctly identified and rapidly eliminated, due to the fact that there is more than one mycobacterial strain that can be detected through microscopic Ziehl–Neelsen examination [also called sputum acid-fast bacilli (AFB) test], but only one generates chronic PTB (8, 9). In other words, sputum microscopy, although fast and inexpensive, is characterized by low sensitivity (61%), therefore involving a low detection rate of *M.tb*. infection (7, 8). A sputum-smear negative and/or negative culture does not always exclude TB diagnosis and may lead to wrong TB management (7–9); this accelerated the use of Xpert Gene MTB/RIF automated rapid molecular assay, which is less sensitive than culture (92%), as a more sensitive method than sputum-smear microscopy for fast identification of PTB as well as rapid assessment of rifampicin susceptibility (6, 10, 11). However, available diagnostic tests and mycobacterial cultures are rather time-consuming compared to point-of-care tests and also require both logistical measures and experienced personnel in order to properly diagnose TB (12, 13). Although GeneXpert MTB/RIF assay is preferred as a diagnostic tool in HIV-positive individuals due to higher sensitivity compared to smear microscopy and faster results compared to mycobacterial culture (14), it imposes various demands such as constant connection to electricity, proper temperature flow, and dedicated personnel to ensure installation functionality in comparison with rapid serological assays (13, 15). Even more, following WHO recommendations to maximize case findings, preclinical evaluation is also based on symptom screening (WHO 4-SS: the presence of at least one of the following in the last 30 days: cough, fever, night sweats, or weight loss), characterized by high sensitivity, but reduced specificity, hence low effectiveness in evaluating TB (8, 11, 16). Cicacci et al. draw attention that screening HIV-positive patients through the WHO-4-SS method may lead to more than 22% of missed TB cases (13), as a significant proportion of *M.tb.*-infected individuals may be asymptomatic (17). Consistently, the specificity of the WHO-4-SS screening method for PTB in HIV-positive patients is approximately 70%, challenging already resource-constrained countries (18). Gersh et al. assessed 0% sensitivity, 87% specificity, and 99% negative predictive value of the WHO-4-SS algorithm, concomitant with 20% sensitivity, 99% specificity, and 99% negative predictive value of the Xpert Gene test for identifying TB in 383 HIV-infected individuals (19). Therefore, the WHO-4-SS screening tool is considered partially effective in HIV-infected patients (17). The most recent point-of-care available triage test detects lipoarabinomannan [lateral flow urine lipoarabinomannan assay (LF-LAM)] in individuals with active TB, as lipoarabinomannan represents an essential lipopolysaccharide for mycobacterial cellular wall (17). LF-LAM has multiple advantages: cost-effectiveness, involves a simple procedure, does not require special equipment, provides rapid results, and high specificity in HIV-infected patients (20). However, LF-LAM presents low sensitivity (56% in patients with CD4 count ≤ 100 cells/μl and 26% in patients with CD4 count > 100 cells/μl), and its specificity decreases proportionally with higher CD4 counts in severe HIV infection (13, 20). Nevertheless, increased sensitivity values for a screening test would rule out TB in non-infected individuals and would also limit the use of more expensive confirmatory tests, as the ones mentioned above, in patients exposed to a higher risk of *M.tb.* infection (thus with increased specificity) (10, 11, 14). Therefore, in order to control TB burden, rapid and reliable screening strategies are imperiously necessary, especially in African regions with an increasing number of HIV-positive patients in the last years (13).

Initiation of anti-TB pharmacotherapy should be approached in suspected but unconfirmed cases as treatment delays may increase transmission burden and may lead to poorer outcomes, especially in patients with immunosuppression (21). In case of HIV infection—the most important risk factor in developing active TB (22)—as soon as antiretroviral therapy is administered, the mortality rate decreases and the prognosis is improved (3, 21, 23). This particular need of early bacterial identification is increased by low-adherence antituberculotic regimens: first-line agents represented by isoniazid, rifampicin, pyrazinamide, ethambutol, and streptomycin (mostly used in primary active TB), and second-line agents (mostly used in resistant bacilli strains) (5, 24–26). First-line regimens are recommended as a directly observed therapy (DOT) in drug-susceptible TB in individuals residing in settings with a low proportion of resistant strains for 6 months as follows: intensive phase (rapid mycobacterial reduction) when agents are administered daily for 2 months and continuation phase (sterilization phase with isoniazid and rifampicin given thrice every week) for the next 4 months (21, 24, 27). In case of isoniazid or rifampicin resistance, an expanded second-line regimen is recommended for more than 12 months, which includes a fluroquinolone (levofloxacin or moxifloxacin) or an injectable agent (such as amikacin, kanamycin, or capreomycin), repurposed drugs (clofazimine or linezolid), or the most recent approved agents (bedaquiline or delamanid) (23, 27). Moreover, continuation phases can be expanded in case of extrapulmonary TB; however, the regimens are similar to the ones used in PTB (27). Recent growth in both multi-drug-resistant TB forms (MDR-TB—due to strains resistant to rifampicin and isoniazid) and extensively drug-resistant TB forms (XDR-TB—due to additional resistance to second-line agents) is caused by the improper use of antibiotics and difficult diagnosis procedures that require even more time than primary active TB, thus urging the necessity to rapidly and specifically diagnose bacterial TB strains (6, 28, 29).

The particularity of PTB lies in the immunological fight between *M.tb.* and the host, based on the interaction of bacterial strains and inflammatory biomarkers released by macrophages, monocytes, neutrophils, and lymphocytes (30–34). C-reactive protein (CRP) is also a non-specific biomarker in TB, with highly increased plasmatic concentrations, due to sputum bacillary load and severity of inflammation (30, 35). Human liver CRP production usually forgoes clinical symptoms (30, 31). CRP can be measured semi-quantitatively using capillary blood or quantitatively from either venous or capillary blood through different immunoturbidimetric methods and rapid cost-effective quantified point-of-care tests (POC-CRP assays) that provide results in less than 5 min (31, 36–38). Moreover, POC-CRP tests are easily interpretable by specialists and available worldwide in comparison with reference diagnosis standards (12). On the other hand, in order to correctly implement POC-CRP tests, the program requires a valid quality method, trained certified applicants, and continuous internal control based on distributors’ manuals (38). Although CRP is a non-specific biomarker in inflammatory diseases, researchers have noted elevated levels associated with other bacterial infections, burns, traumas, rheumatic diseases, and various carcinomas and metastatic stages in lung, breast, digestive, hepatic, and ovarian tissues (39). More recently, CRP/albumin ratios have been proven to be a powerful mortality prognosis marker in hypertensive COVID-19 adults (40), and systematic research should be performed in order to assess the screening and diagnostic accuracy of this serologic marker.

In the past years, different types of studies have been published (retrospective, comparative, multi-center, and clinical trials) that claim the use of CRP as a TB screening test for TB (both pulmonary and extra-pulmonary), in various ethnicities, with several comorbid pathologies (12, 41–45). This increased interest in CRP research has prompted us to evaluate through statistical analysis if CRP is an adequate screening tool, as a rule-out test. Recent literature has shown that TB screening could be intensified and improved by using plasmatic CRP concentrations, especially in low-income countries due to the cost-effectiveness of this biomarker analysis (11, 41). Nevertheless, CRP has also been proposed as a solid candidate for TB screening in HIV-positive patients, providing prognostic values and leading to a more productive disease management (37, 46). Shapiro et al. also underlined the importance of CRP as a discrimination factor between culture-positive and culture-negative specimens (47). Moreover, CRP proved higher accuracy and specificity when evaluated as a TB case-finding test in comparison with WHO-4-SS (12, 47). Although CRP does not identify drug resistance, its potential clinical relevance as a screening test in PTB patients and as a reliable tool in monitoring treatment outcomes justifies the concept of our study (48).

The objective of this study is to determine the accuracy of the use of CRP as a screening biomarker for TB in adults with HIV infection. Our further question refers to the clinical cutoff point(s) of CRP that could indicate a significant inflammation and predicts the presence of PTB in HIV-infected patients. A previous meta-analysis found CRP as a considerable promising tool to ease systematic screening for active TB (49). Since this previous meta-analysis, new studies have been published. WHO promotes intensified TB case identification in HIV-positive adults by WHO 4-SS; thus, we investigated whether rapid CRP tests are more valuable than WHO 4-SS (8, 49, 50). In order to determine the pooled sensitivity and specificity of the CRP test for PTB in outpatients with HIV infection, we performed a meta-analysis update with other subgroups. We planned to conduct a subgroup analysis for CRP cut-points where sufficient data were available.

## Methods

### Search strategy

This meta-analysis was conducted in accordance with the PRISMA (Preferred Reporting Items for Systematic Reviews and Meta-Analyses) statement. We searched MEDLINE, Web of Science, and SCOPUS, until 19 October 2021, following terms (“tuberculosis” AND “C-reactive protein”) OR (“tuberculosis” AND “CRP” AND “ screening test” AND “diagnosis”). The study identification also included manual search, with the screening of the citations of the relevant studies. Two review authors (A-D.M. and M-S.S.) independently extracted data using Excel to determine potentially eligible studies. The disagreements were resolved through discussion and, if necessary, consulting a third review author (A.T-S.).

### Study selection

Inclusion criteria to identify studies that directly address the research question were carefully defined: patients were limited to ambulatory patients because hospitalized patients may have different acute inflammatory conditions, other than HIV infection conclusive for our study that could influence the CRP level. The inclusion criteria were as follows: (a) randomized clinical trials investigating the CRP in HIV-positive active PTB in adults; (b) studies including HIV-positive patients with symptoms or reactivated manifestations of PTB; (c) studies including HIV-positive patients who have not previously been on antitubercular treatment; (d) studies consisting of original articles, peer-reviewed with randomized controlled trials that evaluated the use of CRP, by examining sensitivity and specificity; (e) studies written in English; and (f) studies including mycobacterial reference standard or/and a composite reference standard diagnosis. Eligible studies in which CRP was measured through quantitative laboratory-based and POC assays were also considered. We only included studies that reported data comparing the index test(s) to an acceptable reference standard from which we could extract true positive (TP), true negative (TN), false positive (FP), and false negative (FN) values. The included studies were selected after reviewing the abstract and full text for eligibility.

We included all published manuscripts that primarily assessed CRP levels marking the presence of PTB and also the gold standard diagnosis criteria for TB. Studies that mentioned GeneXpert MTB/RIF assay or WHO score, sustained by radiographic evidence as diagnosis methods for PTB, were also considered for analysis.

We excluded studies that (a) measured CRP through non-quantitative methods or not measured CRP; (b) lacked CRP cutoff values; (c) discussed comorbid inflammatory conditions in patients without HIV condition; (d) diagnosed TB through methods based on inadequate standard reference; (e) included patients with extrapulmonary TB or other pulmonary infections determined by a different strain of mycobacteria; (f) included children; (g) were written in a language other than English; and (h) contained data insufficient to easily distinguish between TP and TN cases. If we needed more information (for example, TP, TN, FP, and FN at 8 mg/L threshold for CRP), we contacted primary study authors for it. The target condition was active PTB in HIV-infected patients; thus, we excluded the studies that also involved patients with extrapulmonary TB that cannot be separated.

### Data extraction and risk of bias assessment

We appraised the quality of studies using the Quality Assessment of Diagnostic Accuracy Studies-2 (QUADAS-2) tool, which consists of four domains: patient selection, index test, reference standard, and flow and timing (differential verification of TB status for study participants). All these domains were assessed for risk and bias.

### Statistical analyses

We performed this meta-analysis to estimate pooled sensitivity and specificity using a bivariate random-effects model and a Bayesian approach. Random-effects models were chosen to describe the variability in test accuracy across studies.

The TP, FP, TN, and FN values were extracted from the included studies or the studies’ correspondence authors were contacted to provide us with this information. We presented individual study results graphically in forest plots, by plotting the estimates of sensitivity and specificity [95% confidence interval (CI)].

Exploratory analyses were undertaken in Review Manager 5 (RevMan 5), and we used R for the definitive analyses. The R packages INLA and mada were used for the meta-analysis of diagnostic accuracy. The bivariate model provided a summary receiver operating characteristic (SROC) curve that integrated receiver operator characteristic curves of primary studies. We calculated area under the curve (AUC) and partial AUC for every SROC plot.

We grouped the studies evaluating CRP by the threshold of 8 mg/L and 10 mg/L. Mycobacterial culture (solid or liquid) or composite reference standards or bacterial microscopic examination through the Ziehl–Neelsen method and fluorescent method was used as reference standard. We investigated the key parameters of summary ROC curves and summary sensitivity–specificity points.

Heterogeneity was investigated through visual examination of forest plots and ROC plots of the raw data. Descriptive statistics included the pooled sensitivity, pooled specificity of the studies, their diagnostic odds ratio (DOR, a measure of the effectiveness of the diagnostic test; higher DOS indicates better test performance), Higgins *I* ^2^ (assess the consistency of the results of studies in meta-analysis: a value of 0% indicates no observed heterogeneity, and larger values show increasing heterogeneity), and Cochran’s *Q* statistic. We performed *χ* ^2^ test to assess heterogeneity of sensitivities and specificities, the null hypothesis being, in both cases, that all are equal for all the studies. The significance level was 0.05.

Sensitivity analyses were performed by limiting inclusion in the meta-analysis to the studies, for example, that scored “yes” for the QUADAS-2 question “Did the study avoid inappropriate exclusions?”, which expresses a low risk of bias for participant selection, or, another example, the studies that scored “yes” for the QUADAS-2 question “Is the reference standard likely to correctly classify the target condition?”, which leads to a low risk of bias for the reference standard.

## Results

### Search results

**Figure 1:**
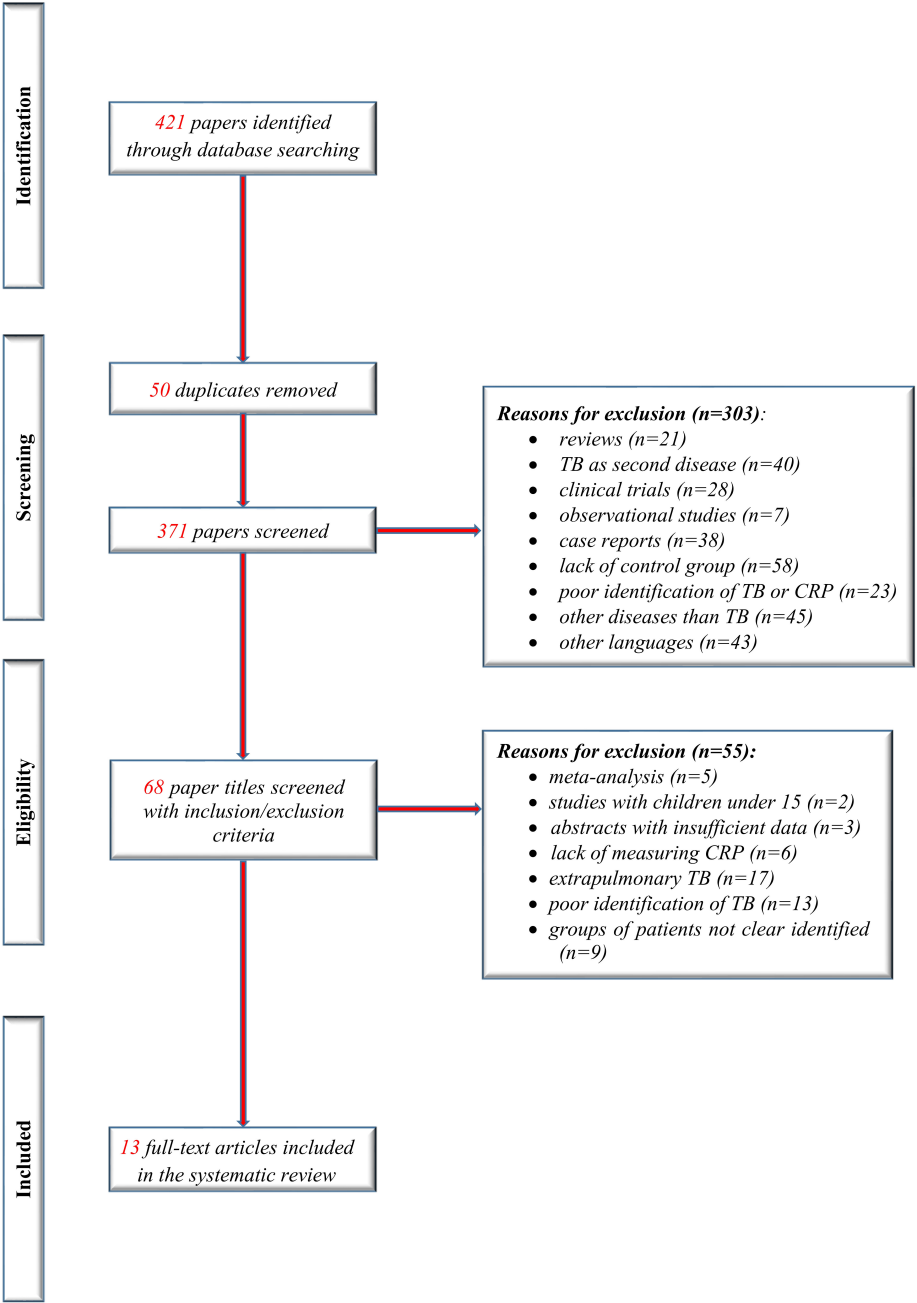
Study flow PRISMA diagram.

We identified 13 studies that met the inclusion criteria of our present study (eight new studies since the previous review). **Figure 1** shows the flow of studies in the review, with the steps of the study selection process in a PRISMA diagram.

All included studies were performed in countries with a high TB/HIV burden, classified as low-income or middle-income countries (World Bank 2020) as described in **Table 1** with the other characteristics of included studies.

**Table 1:** Characteristics of included studies.

Study (name, year)	Country	Number, type of patients and median age	Methods of TB screening and diagnostic (*reference standard diagnostic test)	CRP dosing method (assay kit/analyzer type)
Ciccaci 2019 (41)	Mozambique, South Africa	*n* = 143 (21 HIV-positive/Xpert-positive + 122 HIV-positive/Xpert-negative), median age = 36	WHO 4-SS + *Xpert Gene MTB/RIF (Assay system, Cepheid, Sunnyvale, CA, USA)	Measured before anti-TB treatment through enzyme-linked immunosorbent assay—Human CRP ELISA—kit (Arigo Biolaboratories Corporation, Hsinchu City, Taiwan)
Drain 2014 (50)	KwaZulu-Natal, South Africa	*n* = 93 (45 HIV-positive/TB-positive—from which 37 were culture-positive and 8 were SSM-positive—and 48 HIV-positive/TB-negative), median age = 35	*Culture (both liquid BACTEC TB 960 systems and solid) + SSM (both Ziehl–Neelsen and auramine fluorescent staining) + WHO 4-SS + chest radiography evaluation	Measured before anti-TB treatment through both immunoturbidometric assay with Dimension RXL analyzer from Dade-Behring (Deerfield, IL, USA—laboratory-based high-sensitivity method) and immunoassay POC (NycoCard CRP test—Axis-Shield, Oslo, Norway)
Lawn 2013 (46)	Cape Town, South Africa	*n* = 496 (81 HIV-positive/culture-positive + 415 HIV-positive/culture-negative), median age = 33.6	SSM (fluorescent microscopy) + *culture (liquid BACTEC MGIT) + WHO 4-SS + Xpert Gene MTB/RIF + chest radiography evaluation	Measured before anti-TB treatment through enzyme-linked immunosorbent assay—Quantikine (R&D Systems Inc., Minneapolis, MN, USA)
Olsson 2019 (16)	Ethiopia, East Africa	*n* = 260 (130 HIV-positive/PTB-positive + 130 HIV-positive/TB-negative), median age = 33.6	*SSM + *culture (liquid) + WHO 4-SS + *Xpert Gene MTB/RIF (Cepheid, Sunnyvale, CA)	Measured before anti-TB treatment through immunoturbidometric assay (Bio-Rad Laboratories, Hercules, CA—Bio-Plex 200 reader) and Magnetic Luminex Assay (R&D Systems Inc., Minneapolis, MN)
Shapiro 2018 (47)	Durban, South Africa	*n* = 425 (42 HIV-positive/PTB culture-positive + 383 HIV-positive/PTB culture-negative), median age = 32	*Culture (liquid BACTEC MGIT systems) + WHO 4-SS + SSM + Xpert MTB/RIF + chest radiography evaluation	Measured before anti-TB treatment through immunoturbidometric assay through Roche Integra analyzer (Mannheim, Germany)
Wilson 2006 (51)	South Africa	*n* = 130 (105 HIV-positive/TB culture-positive or histological features and detection of any mycobacterial specimen and 25 HIV-positive/possible TB with anti-TB-treatment response), median age = 34.4	SSM (auramine-rhodamine fluorescent microscopy) + *culture (liquid BACTEC MGIT) + chest radiography evaluation	Measured before anti-TB treatment and also assessed at weeks 2, 4, and 8 after anti-TB treatment through immunoturbidometric assay (Beckman Coulter CX7)
Wilson 2011 (52)	KwaZulu-Natal, South Africa	*n* = 200 (112 HIV-positive/TB culture-positive and 88 HIV-positive/extraPTB or both PTB and extraPTB), median age = 34.4	*SSM (fluorescent microscopy) + *culture (liquid BACTEC MGIT) + WHO 4-SS + chest radiography evaluation	Measured before anti-TB treatment or after maximum one week of anti-TB treatment through immunoturbidometric assay (Olympus AU640 and Dade Dimension RXL)
Yoon 2014 (37)	Mbarara, Uganda	*n* = 201 (5 HIV-positive/PTB-positive and 196 HIV-positive/PTB-negative), median age = 33	*SSM + *culture + WHO 4-SS	Measured before anti-TB treatment through immunoassay point-of-care (iCHROMA POC-CRP Reader, BodiTech Med Inc., South Korea)
Yoon 2017 (49)	Kampal, Uganda	*n* = 1,177 (163 HIV-positive/PTB culture-positive, from which 84 PTB also confirmed with Xpert Gene MTB/RIF positive and 1,014 HIV-positive/PTB-negative), median age not mentioned	SSM (Capilia TB, TAUNS, Japan or MPT64 assay, Standard Diagnostics, South Korea) + *culture (solid Löwenstein-Jensen and/or liquid BACTEC MGIT 960) + WHO 4-SS + Xpert Gene MTB/RIF (Cepheid USA)	Measured before anti-TB treatment through immunoassay point-of-care (iCHROMA POC-CRP Reader, BodiTech Med Inc., South Korea)
Boyles 2020 (18)	Johannesburg, South Africa	*n* = 207 (75 HIV-positive/TB-positive + 132 HIV-positive/TB-negative), median age = 36	WHO-4-SS + SSM + *culture (liquid–mycobacterial growth indicator tube, MGIT BACTEC 960 TB System) + Xpert MTB/RIF Ultra (Ultra)	Measured before anti-TB treatment through point-of-care method (Abbot Afinion AS100 analyzer)
Gersh 2021 (19)	Western Kenya	*n* = 383 (5 HIV-positive/TB culture-positive + 378 HIV-positive/TB-negative), median age = 37	WHO-4-SS + SSM (fluorescence microscopy and AFB) + *culture (commercial broth method, MGIT Manual Mycobacterial Growth System, Becton-Dickinson, Franklin Lakes, NJ) + Xpert Gene MTB/RIF (Xpert, Cepheid, Sunnyvale, CA)	Measured before receiving anti-TB treatment, using a high-sensitivity assay (Cobas Integra 400 Plus (Roche Diagnostics, Rotkreuz, Switzerland)
Mwebe 2021 (53)	Kampala, Uganda	*n* = 605 (103 HIV-positive/TB-positive + 502 HIV-positive/TB-negative—confirmed with TB if at least one sputum culture was positive), median age = 34	Xpert Gene MTB/RIF (Cepheid USA) + *liquid mycobacterial culture (BACTEC MGIT 960) + WHO-4-SS	Measured before anti-TB treatment through standard point-of-care assay from capillary blood (iCHROMA POC-CRP Reader, BodiTech Med Inc., South Korea)
Samuels 2021 (14)	South Africa, Cambodia, Peru, Georgia, Vietnam	*n* = 765 (111 HIV-positive/TB culture-positive + 274 HIV-negative/TB culture-positive + 102 HIV-positive/TB-negative + 253 HIV-negative/TB-negative + 6 unknown HIV status/TB-positive + 19 unknown HIV status/TB-negative), median age = 36	SSM (fluorescence microscopy with auramine staining) + *culture (liquid MGIT Becton Dickinson, Franklin Lakes, USA + solid Lowenstein-Jensen medium) + Xpert Gene MTB/RIF (Cepheid, Sunnyvale, USA)	Measured before anti-TB treatment through a latex immunoassay (Multigent CRP Vario assay on Abbott Architect C8000)

TB, tuberculosis; CRP, C-reactive protein; WHO-4SS (4-symptoms screening): symptom screen positivity is defined by the presence of any current cough, fever, night sweats, or weight loss in the previous 30 days; SSM (sputum-smear microscopy): identification of AFB (acid-fast bacilli) through the Ziehl–Neelson method or the auramine fluorescent method; culture: can be realized through solid medium (Löwenstein–Jensen) and/or the liquid BACTEC MGIT (Mycobacterial Growth Indicator Tube) 960 system.

### Bias assessment

**Figure 2:**
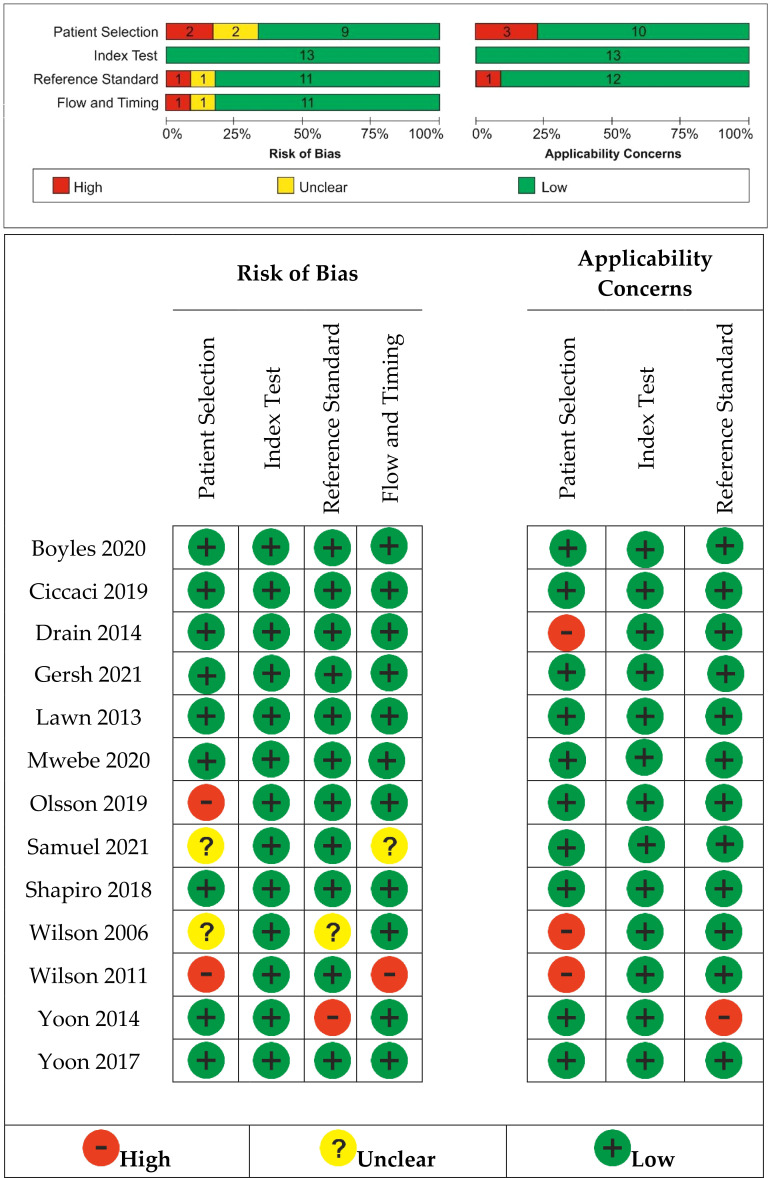
Risk of bias and applicability concerns summary: review authors’ judgments about each study.

The risk of bias and applicability concerns is shown in **Figure 2**. Most studies had a low risk of bias.

We found an unclear risk of bias in patient selection for Samuels (2021) (14), a retrospective case–control study with no details on how the allocation list was concealed, and Wilson (2006) (51), where it was unclear if patients’ randomization was performed. Olson (2019) (16) and Wilson (2011) (52) were considered at high risk of bias selection because the random sequence generation was not included into the patients’ selection.

Yoon (2014) (37) was considered to have a high risk of bias for reference standard because the reference standard, its conduct, or its interpretation could have introduced bias and also raised concerns regarding the applicability of the reference standard. In Wilson (2006) (51), it was unclear if the reference standard results were interpreted without knowledge of the results of the index tests.

Samuels (2021) (14) did not clearly explain if the patients received the same reference standard. Wilson (2011) (52) introduced a risk of bias through flow and timing because not all patients were included in the analysis; some of them were not able to attend for regular review.

High applicability concerns were raised for patient selection because the included patients and setting could not match the review question in the case of three studies (50–52).

### The pooled results

Thirteen studies were included in the meta-analysis that evaluated CRP for PTB among HIV outpatients (14, 16, 18, 19, 41, 46, 47, 49–54). The studies provided data for 4,355 HIV-positive adults, including 891 (20%) with PTB.

For the CRP threshold of 10 mg/L, sensitivity estimates range between 20% and 98%, and specificity estimates range between 26% and 96%. Twelve studies including 3,751 patients were included in the diagnostic meta-analysis.

**Figure 3:**
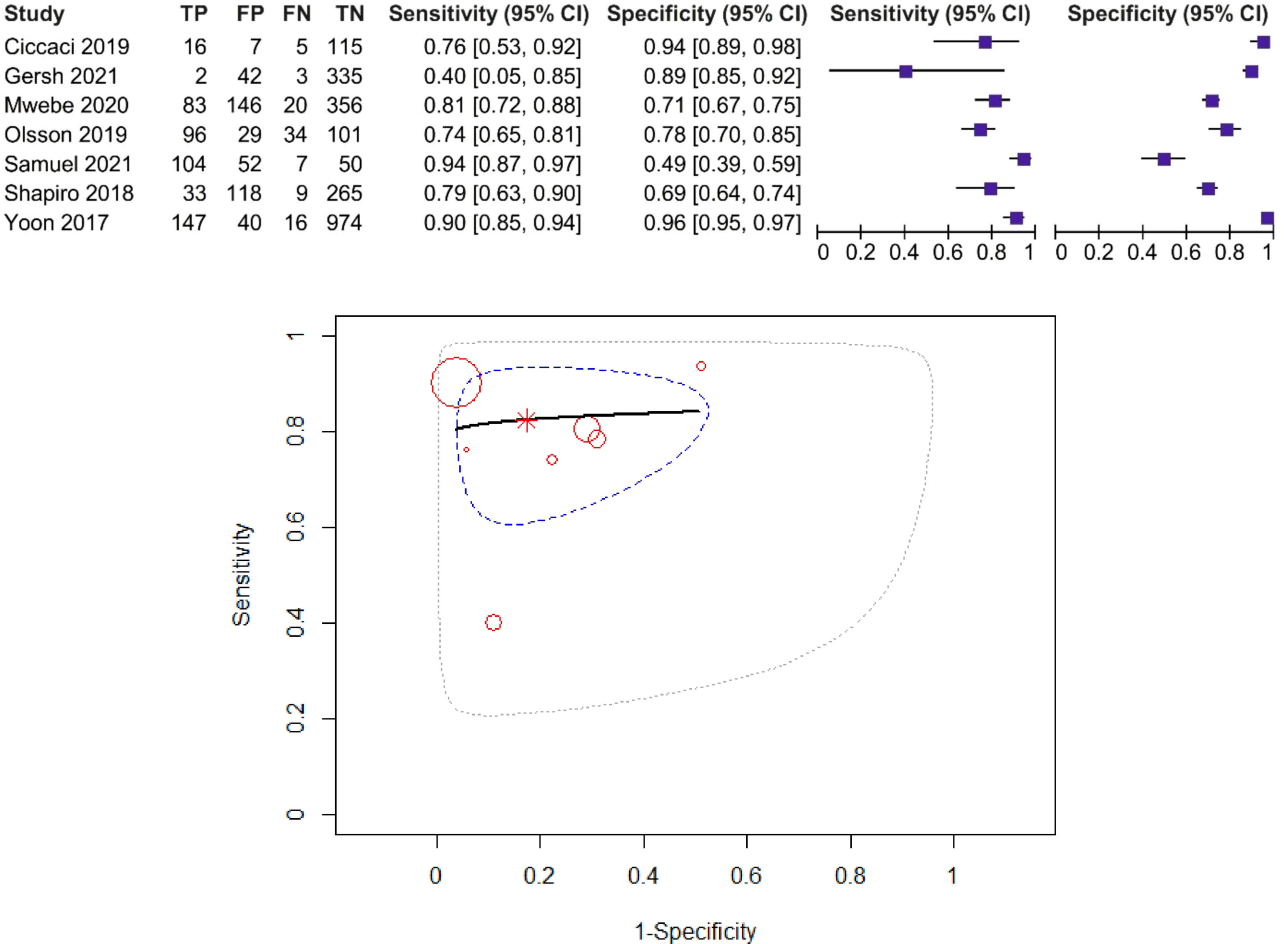
Forest plot and SROC curve (HIV patients, using the CRP threshold of 10 mg/L). The symbol * represents the pooled sensitivity and specificity.

The forest plot and SROC curve of CRP sensitivity and specificity for PTB for studies among HIV-positive patients, using the CRP threshold of 10 mg/L, are given in **Figure 3**. The dotted blue curve represents the 95% confidence region and the dotted closed curve represents the 95% prediction region.

The pooled sensitivity was 87% (95% CI: 76%–93%) and the pooled specificity was 67% (95% CI: 49–81%), test for heterogeneity *I* ^2^ = 29.49%, *Q* = 12.77, *p* = 0.174, and DOR = 13.26. The *χ* ^2^ test (*p* > 0.05) and *I* ^2^ < 50% suggested non-significant heterogeneity of sensitivities and specificities, so we used fixed-effects meta-analysis. AUC and partial AUC were 0.858 and 0.841, respectively.

**Figure 4:**
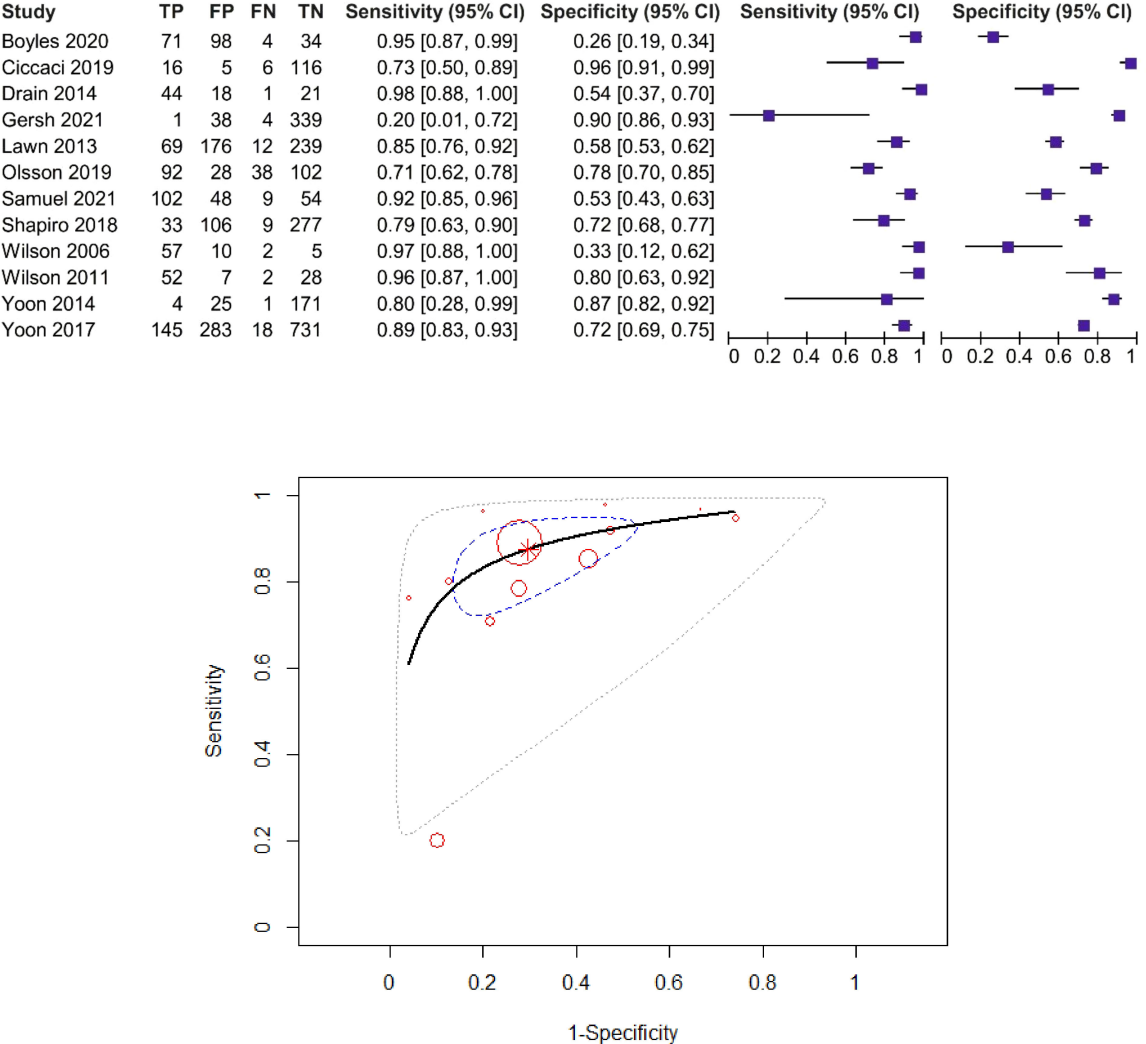
Forest plot and SROC curve (HIV patients, using the CRP threshold of 8 mg/L). The symbol * represents the pooled sensitivity and specificity.

For the CRP threshold of 8 mg/L, sensitivity estimates range between 40% and 94%, and specificity estimates range between 49% and 96%. Seven studies including 3,205 patients were included in the diagnostic meta-analysis (14, 16, 19, 41, 47, 49, 54). The forest plot and SROC curve of CRP sensitivity and specificity for PTB for studies among HIV-positive patients, using the CRP threshold of 8 mg/L, are presented in **Figure 4**.

The pooled sensitivity was 82% (95% CI: 72%–89%) and the pooled specificity was 82% (95% CI: 67%–92%), test for heterogeneity *I* ^2^ = 0%, *Q* = 4.95, *p* = 0.55, and DOR = 19.12 (95% CI: 6.5–56.1). The *χ* ^2^ test (*p* > 0.05) and *I* ^2^ < 50% suggested non-significant heterogeneity of sensitivities and specificities, so we used fixed-effects meta-analysis. AUC and partial AUC were 0.879 and 0.833, respectively.

## Discussion

This meta-analysis updates the current literature, including 13 studies, on the accuracy of the CRP screening test for PTB in HIV-positive adults (14, 16, 18, 19, 41, 46, 47, 49–54). The pooled estimates for sensitivity and specificity did not remain similar using a CRP cut-point of 10 mg/L, compared to the current review (pooled sensitivity = 87% and pooled specificity = 67%) and prior review (pooled sensitivity = 82% and pooled specificity = 82%), respectively (48).

We chose to evaluate the accuracy of the CRP screening test separately for studies that present the results for the threshold of 10 mg/L vs. 8 mg/L because the SROC curves do not estimate with respect to the identification of points on the curve that show a particular threshold. The pooled sensitivity was 86% in the case of the 10 mg/L threshold and 81% in the case of the 8 mg/L threshold. Better pooled specificity was found in the case of the 8 mg/L threshold: 88% vs. 73% in the case of the 10 mg/L threshold.

As WHO recommends, people infected with HIV or living with HIV should be systematically screened for active TB through WHO-4SS assessment or chest radiography evaluation as a second screen test (8, 55, 56). Further clinical diagnostics is established by different algorithms: mycobacterial culture, SSM, and Xpert MTB/RIF test (8, 56). Although culture is the gold standard for TB diagnosis, it is not usually approached as an initial test, due to the longer time required for results (2 to 6 weeks) (8, 56). Thus, in poor-resource and HIV high-prevalence areas, diagnostic algorithms include SSM and Xpert MTB/RIF that provide final results in less than 24 h (8, 56). Even more, a good TB diagnostic test must have at least 90% sensitivity and 70% specificity, and CRP has been proven to be the closest parameter to meet WHO recommendations (14, 53, 56). Deficiency in screening strategies could lead to delayed TB diagnosis or misdiagnosis, higher rates of transmission and mortality, with disastrous financial consequences (8, 56). Various clinical algorithms have been developed and assessed for management of TB; however, these present shortcomings in HIV-positive patients (42, 45). Recent studies suggested that CRP presents higher specificity values and a better performance for the identification of active PTB than WHO symptom screening strategies (16, 46, 55). The meta-analysis conducted by Yoon and collaborators also proved high values (93%) of sensitivity and moderate values of specificity (63%) for CRP as a screening method among individuals with PTB-specific symptoms (49). Samuels et al. included both cutoff points for CRP and obtained even better values for sensitivity and specificity compared with culture diagnostic methods as CRP concentrations decreased: 91.9% sensitivity and 52.9% specificity for a cutoff point of 10 mg/L, and 93.7% sensitivity and 49% specificity for a cutoff point of 8 mg/L (14). Mwebe et al. obtained 81% sensitivity and 71% specificity for CRP diagnostic accuracy when compared to culture methods and confirmed the utility of CRP as a TB screening tool for HIV-positive individuals (53). The maximum value for CRP sensitivity extracted from the included studies in our meta-analysis was 95% at a cutoff point of 10 mg/L, but correlated with 26% specificity compared to sputum tests; the authors characterized CRP as an important predictor for TB (18). Researchers also mentioned that CRP became less specific while increasing sensitivity values proportionally with a larger number of symptoms: 93.5% sensitivity and 37% specificity for CRP > 8 mg/L, and 92.5% sensitivity and 41.4% specificity for CRP > 10 mg/L (14). In accordance with WHO recommendations, when CRP is used as a screening test and provides positive results, individuals should undergo evaluation through specific diagnostic methods (14). CRP sensitivity reached higher values than 90% in cases of TB diagnosed through Xpert Gene test; therefore, CRP usage could provide cost-effectiveness as it would reduce about 40% the need to use Xpert Gene as a diagnostic method (14). Moreover, the excellent sensitivity of CRP appeared in HIV-positive individuals, but with possible insufficient specificity, raising the need to adjust even more cutoff points for CRP in order to improve screening performance (14, 18). In line with this, our results demonstrate that CRP is an adequate screening test in regions with high prevalence of HIV infections. On the other hand, it is important to note the high quality for retrieved studies with a low risk of bias for the QUADAS‐2 domains of participant sampling, index test, reference standard, and flow and timing for our research aim.

Although recent research indicates the use of immunological marker CRP as specific enough for distinguished TB diagnosis, this acute-phase protein can be especially relevant for monitoring the severity of the disease or the effectiveness of the treatment (41, 49, 50, 52). Along other host serum proteins such as alpha-2-macroglobulin, haptoglobin, fibrinogen, complement factor H, serum amyloid P, and transthyretin, CRP has recently been studied as a biosignature and point-of-care screening test for TB in HIV-infected patients from African settings (15). Fuster et al. analyzed the association between pro-inflammatory biomarkers (CRP, TNF-α, IL-6, IL-10, serum amyloid A, cystatin-C, and monocyte chemotactic protein-1) and mortality rates in HIV-diagnosed individuals and obtained positive statistical correlation (57). CRP can be instantly POC measured, saving time and without posing economic burden in comparison with TB symptom screening test or other molecular tests (41, 46, 49, 55). On the other hand, the faster PTB is diagnosed, the lower severity and mortality caused by this infection, especially in high-risk groups (such as HIV-infected patients) (16, 44, 46, 49, 58).

HIV-infected adults are disproportionately influenced by *M.tb.*, due to higher FN rates, lower sensitivity, and difficult accessibility in TB-endemic regions of screening tests (41, 42, 44, 47, 50). In high-burden areas, HIV infection deteriorates immune functions by lowering CD4^+^ T cells while the increasing risk of primary *M.tb.* infection or reactivation in case of latent TB (22). HIV-infected individuals are 26 times more likely to be diagnosed with TB in comparison with those non-HIV infected (59). As *M.tb.* is characterized by an intriguing ability to adapt and survive on long term within the host even in case of immune responses and cytokine activation (60), HIV infection leads to deficiency in immune response ensuring a proper environment for TB development (22). Skogmar et al. found an inverse correlation between CRP and neopterin levels and CD4 cell count (61); in other words, increased inflammatory responses and immune activation are correlated with CD4 lymphocytopenia in adults infected with *M.tb* (60, 61). A superior inflammatory response and increased frequency of dissemination have been noticed in HIV-positive TB patients in comparison with non-TB patients; thus, even though CRP has shown insufficient values of sensitivity and specificity in screening for TB, rapid CRP test seems promising for exclusion of PTB in HIV-positive patients (41, 42, 46, 50). As shown, this could further facilitate differential diagnosis that could lead to rapid antiretroviral therapy and mortality reduction (46, 50). Moreover, Cicacci et al. confirm that higher bacillary load implies higher CRP levels and underlines a better specificity value of CRP than WHO symptom screening (16, 46, 49) or other cross-examined plasmatic inflammatory markers (16, 58). Other studies mentioned the importance of serum-analyzed CRP not only as a cost-effective method itself, but also as a potential test for reducing the employment of other molecular tests (16, 46, 49). Patients who had higher CRP values were more likely to be SSM-positive or detected by the GeneXpert MTB/RIF assay, which accentuated the prognostic utility of CRP as a potential screening PTB test (14, 16, 46, 53, 54).

The importance of establishing a threshold for CRP has also been mentioned, but some researchers emphasize that increasing the values of threshold (for example, from 5 to 10 mg/L) decreases, even more, the test sensitivity (with more than 10%) (47, 49, 54). The closest cut-point to WHO recommendations was obtained from the research conducted by Gersh et al.: CRP > 3.3 mg/L (sensitivity 80%, specificity 72%), while the cutoff point 10 mg/L led to decreased values for sensitivity (20%) concomitant with increased specificity (90%) (19). In other words, raising the analyzed CRP value could lead to a higher number of TB cases with improper prognostication (47), if other tests are not performed. CRP seems not to be conclusive as a singular TB diagnosis marker, but successful in facilitating systematic TB screening, when associated with the gold standard reference method or GeneXpert assay, within HIV-positive groups (49). This is the reason why the evaluation of CRP as a screening biomarker for active PTB has been widely analyzed and argued by researchers in the past years (11, 41, 47, 49, 52, 54), especially since the majority of the infected individuals, children, and adults are diagnosed with PTB (62–64).

WHO recommends the GeneXpert ^®^ MTB/RIF assay as the initial diagnostic test in adults and children with presumed HIV-associated TB, rather than conventional microscopy and culture (1, 2). The LoopampTM MTBC Detection Kit is also recommended by WHO as a rapid diagnostic test to detect TB among people with signs or symptoms of TB (1). There is still no single rapid, accurate, and robust TB diagnostic test appropriate for use at the point of care (1), although diagnostics and reducing the time to introduce an adequate therapy are top priorities for WHO (56). Only the urine-based LF-LAM test was recommended in combination with existing TB tests to increase early TB diagnosis and treatment (1). WHO mentions sensitivity and specificity values for diagnostic methods as follows: 100% and 100% for liquid gold standard culture, 92% and 99% for Xpert Gene test, 61% and 98% for conventional sputum-smear microscopy, and 24% and 94% for chest radiography after negative sputum test or Xpert Gene test (8); our meta-analysis confirms 82% sensitivity and 82% specificity for CRP at 8 mg/L, underlining its accuracy as a screening strategy for active TB cases among HIV-positive patients.

Our research has several limitations. Most significantly, the reference test characteristics in this meta-analysis were not common across all studies, which is an important source of heterogeneity. Thus, there is no strong reference standard. The different reference standards among the article represent a source of potential bias. We have tried to separately evaluate different reference standards in order to predict the level of sensitivity and specificity compared to the gold standard reference, but the included studies did not present sufficient data regarding the culture test. The different reference standards could have facilitated for a trade-off between yield of TB screen test and participants included in each analysis.

The outcome of this study could also have introduced bias due to the heterogeneous patient population or study design. For example, we evaluated the study conducted by Wilson et al. (2011) that included HIV-positive patients with less than 1 week of antitubercular therapy in comparison with the other studies that included only patients without previous antitubercular treatment. However, a culture conversion often appears after 1 month and up to 2 or 3 months of treatment in PTB active patients (9, 58, 64, 65), underlining that the consistent modifications in CRP values present low probability to appear after only 1 week of antitubercular therapy. Another limitation is that all studies were conducted in sub-Saharan Africa, and most studies were conducted in a single country (South Africa), particularly in settings with a high prevalence of HIV; thus, generalization of findings should be performed with care. This is also one of the reasons for heterogeneity in selected reference diagnostic standards of the included studies.

Although all the studies included in the meta-analysis were all conducted before the COVID-19 pandemic, it is important to mention the involvement of CRP in patients co-diagnosed with TB and COVID-19 with respiratory symptoms similar to those TB diagnosed (66). CRP values were significantly increased in individuals with severe infections, as Bostanghadiri and colleagues noted (66). Moreover, Parker et al. concluded that higher values of CRP were measured in patients diagnosed with all three infections: TB, HIV, and COVID-19 (67). However, researchers recommend CRP as a monitoring and prognosis tool in patients diagnosed with both TB and COVID-19, rather than as a rule-out screening test (68). Multiple investigations are required in order to generalize our results in those individuals.

## Data availability statement

The raw data supporting the conclusions of this article will be made available by the authors, without undue reservation.

## Author contributions

A-DM, AT-S, MB, and M-SS participated in research design and contributed to the data collection. AT-S undertook the statistical analysis and prepared the figures. RC, BSU, and BM interpreted the results from the analysis. A-DM and AT-S wrote the manuscript. BSU and C-GP reviewed the manuscript. All authors read and approved the final manuscript.

## Funding

The Article Processing Charges were funded by the University of Medicine and Pharmacy of Craiova, Romania.

## Conflict of interest

The authors declare that the research was conducted in the absence of any commercial or financial relationships that could be construed as a potential conflict of interest.

## Publisher’s note

All claims expressed in this article are solely those of the authors and do not necessarily represent those of their affiliated organizations, or those of the publisher, the editors and the reviewers. Any product that may be evaluated in this article, or claim that may be made by its manufacturer, is not guaranteed or endorsed by the publisher.
